# Fracture Surgery of the extremities with the intra-operative use of 3D-RX: A randomized multicenter trial (EF3X-trial)

**DOI:** 10.1186/1471-2474-12-151

**Published:** 2011-07-06

**Authors:** M Suzan H Beerekamp, Dirk Th Ubbink, Mario Maas, Jan SK Luitse, Peter Kloen, Taco JM Blokhuis, Michiel JM Segers, Meir Marmor, Niels WL Schep, Marcel GW Dijkgraaf, J Carel Goslings

**Affiliations:** 1 Trauma Unit, Department of Surgery, Academic Medical Center, Amsterdam, the Netherlands; 2 Department of Quality and Process Innovation, Academic Medical Center, Amsterdam, the Netherlands; 3 Department of Radiology, Academic Medical Center, Amsterdam, the Netherlands; 4 Department of Orthopaedic Surgery, Academic Medical Center, Amsterdam, the Netherlands; 5 Department of Surgery, University Medical Center, Utrecht, the Netherlands; 6 Department of Surgery, Saint Antonius Hospital, Nieuwegein, the Netherlands; 7 Department of Orthopedic Surgery, University of San Francisco Medical Center, California, USA; 8 Clinical Research Unit, Academic Medical Center, Amsterdam, the Netherlands

**Keywords:** Fracture, Wrist, Ankle, Calcaneus, Intra-operative imaging, 2D-fluoroscopy, 3D-imaging, Conebeam-CT, 3D-RX

## Abstract

**Background:**

Posttraumatic osteoarthritis can develop after an intra-articular extremity fracture, leading to pain and loss of function. According to international guidelines, anatomical reduction and fixation are the basis for an optimal functional result. In order to achieve this during fracture surgery, an optimal view on the position of the bone fragments and fixation material is a necessity. The currently used 2D-fluoroscopy does not provide sufficient insight, in particular in cases with complex anatomy or subtle injury, and even an 18-26% suboptimal fracture reduction is reported for the ankle and foot. More intra-operative information is therefore needed.

Recently the 3D-RX-system was developed, which provides conventional 2D-fluoroscopic images as well as a 3D-reconstruction of bony structures. This modality provides more information, which consequently leads to extra corrections in 18-30% of the fracture operations. However, the effect of the extra corrections on the quality of the anatomical fracture reduction and fixation as well as on patient relevant outcomes has never been investigated.

The objective of this study protocol is to investigate the effectiveness of the intra-operative use of the 3D-RX-system as compared to the conventional 2D-fluoroscopy in patients with traumatic intra-articular fractures of the wrist, ankle and calcaneus. The effectiveness will be assessed in two different areas: 1) the quality of fracture reduction and fixation, based on the current golden standard, Computed Tomography. 2) The patient-relevant outcomes like functional outcome range of motion and pain. In addition, the diagnostic accuracy of the 3D-RX-scan will be determined in a clinical setting and a cost-effectiveness as well as a cost-utility analysis will be performed.

**Methods/design:**

In this protocol for an international multicenter randomized clinical trial, adult patients (age > 17 years) with a traumatic intra-articular fracture of the wrist, ankle or calcaneus eligible for surgery will be subjected to additional intra-operative 3D-RX. In half of the patients the surgeon will be blinded to these results, in the other half the surgeon may use the 3D-RX results to further optimize fracture reduction. In both randomization groups a CT-scan will be performed postoperatively. Based on these CT-scans the quality of fracture reduction and fixation will be determined. During the follow-up visits after hospital discharge at 6 and 12 weeks and 1 year postoperatively the patient relevant outcomes will be determined by joint specific, health economic and quality of life questionnaires. In addition a follow up study will be performed to determine the patient relevant outcomes and prevalence of posttraumatic osteoarthritis at 2 and 5 years postoperatively.

**Discussion:**

The results of the study will provide more information on the effectiveness of the intra-operative use of 3D-imaging during surgical treatment of intra-articular fractures of the wrist, ankle and calcaneus. A randomized design in which patients will be allocated to a treatment arm during surgery will be used because of its high methodological quality and the ability to detect incongruences in the reduction and/or fixation that occur intra-operatively in the blinded arm of the 3D-RX. An alternative, pragmatic design could be to randomize before the start of the surgery, then two surgical strategies would be compared. This resembles clinical practice better, but introduces more bias and does not allow the assessment of incongruences that would have been detected by 3D-RX in the blinded arm.

**Trial registration:**

Dutch Trial Register NTR 1902

## Background

Fractures of the extremities are common injuries, with an incidence of 38 wrist fractures per 10,000 inhabitants per year,[1] and an estimate of 25,000 - 68,000 ankle fractures per year in the Netherlands [2]. Posttraumatic osteoarthritis can develop after an intra-articular extremity fracture, which can lead to pain and loss of function. According to international guidelines anatomical reduction and fixation are the basis for an optimal functional result [3]. This can be achieved by closed reduction and cast fixation. If a conservative treatment leads to a suboptimal reduction and fixation, surgical treatment is indicated.

During open reduction and internal fixation (ORIF) conventional 2D-fluoroscopy is used to gain more insight in the fracture fragments and fixation material next to a direct view. When it involves complex anatomy or subtle injury 2D-fluoroscopy often underestimates the extent of the injury or the degree of displacement of fracture fragments, which can be misleading to the surgeon. Consequently, postoperative X-ray images and CT-scans frequently show incorrect positioned screws or incongruences, like gaps and step-offs, in the joint surface, while these were not recognized on the intra-operative 2D-fluoroscopic images. E.g. for the ankle and foot even an 18-26% suboptimal fracture reduction is reported [4-6]. More intra-operative information is therefore needed in order to minimize suboptimal fracture reduction.

Recently a 3-Dimensional Rotational X-ray system (3D-RX-system) was developed which can provide more detailed imaging intra-operatively. This system consists of a mobile C-arm unit modified to provide a motorized rotational movement and is combined with a workstation. Next to conventional 2D-fluoroscopy this system can provide multiplanar 3-dimensional reconstruction of the osseous structures.

Several cadaveric studies have been performed on different joints of the upper and lower limb to evaluate the image quality of intra-operative 3D-imaging [7-12]. In these studies 3D-imaging had a better diagnostic value than conventional radiography and 2D-fluoroscopic images. Although the subjective imaging quality was higher in CT-scanning, images of the intra-operative 3D-imaging were comparable in diagnostic value. In addition, in some clinical studies concerning intra-operative 3D-imaging, this modality has shown to provide extra information. Extra corrections after 3D-imaging were performed in 11-30% of the fracture operations [11,13-17]. These corrections concerned suboptimal fracture reduction, like intra-articular steps-offs and fracture gaps, and incorrectly positioned fixation material.

The studies mentioned above have shown that intra-operative 3D-imaging provides additional information and allows the surgeon to recognize problems with fracture reduction and/or fixation during the operation. However, these studies used indirect measurements to establish the added value of intra-operative 3D-imaging. The direct effects on the quality of fracture reduction and fixation and patient relevant outcomes have not yet been investigated.

The aim of this protocol for a randomized clinical trial is to investigate the effectiveness of the the intra-operative use of the 3D-RX-system as compared to the use of conventional 2D-fluoroscopy alone in patients with traumatic intra-articular fractures of the wrist, ankle and calcaneus. This effectiveness will be assessed in two different areas: 1) the quality of fracture reduction and fixation, based on the current golden standard, Computed Tomography. 2) The patient-relevant outcomes like functional outcome, range of motion and pain. In addition, the diagnostic accuracy of the 3D-RX-scan will be determined in a clinical setting and a cost-effectiveness as well as a cost-utility analysis will be performed.

### Choice for the study design

With this study we aim to address multiple issues regarding the intra-operative use of 3D-imaging in fractures of the extremities; the diagnostic value (sensitivity and specificity) of the 3D-RX-scan in a clinical setting, the therapeutical outcome and its effect on the quality of fracture reduction and fixation and the patient relevant outcomes. For this purpose we prefer a blinded randomized design, offering the most robust way to investigate the effect of the intra-operative use of 3D-imaging. In order to compare both techniques, patients will be randomized during surgery after the definitive adjustments on the basis of 2D-fluoroscopy and before 3D-fluoroscopy. This avoids the phenomenon that the surgeon neglects the 2D-fluoroscopy and relies on the envisioned 3D-images for the reduction and fixation later during the surgical procedure. An additional advantage of this design is that both the diagnostic value as well as the effects on the quality of fracture reduction and fixation can be investigated.

A disadvantage of our study design is that half of the patients receive radiation of the 3D-scan, while they cannot benefit from this 3D-scan. Because of the relatively low radiation dose of the 3D-scan, this disadvantage is considered acceptable.

An alternative, more pragmatic, study design would randomize between two operative strategies: with or without intra-operative 3D-imaging. In this design no patient would receive unbeneficial radiation doses and this set-up will probably be a better reflection of the clinical practice. However, comparing two surgical strategies does not allow the assessment of any missed incongruences when using 2D-fluoroscopy alone. In addition, when the surgeon knows he can employ the 3D-imaging strategy, he might change his attitude towards 2D-fluoroscopy and be less accurate. Hence, more bias will be introduced because of the surgeon's attitude towards 3D-imaging. In addition, 3D-imaging may also detect incongruences that are corrected, but may not have any influence on functional outcomes or long-term development of posttraumatic osteoarthritis. This possible overdiagnosing with 3D-imaging cannot be detected with a pragmatic design.

The choice for our study design induced some practical drawbacks, i.e. the blinding of the 3D-scan for the surgeon needs some discipline as the only way to blind the 3D-scan is to turn the screens from the surgeon. In addition this design warrants more administration during surgery because the surgeon evaluates the radiological results after every imaging modality. Because of the use of a secured internet module, this administration is relatively simple.

## Methods/design

### Objectives

The objectives of this randomized clinical trial are to:

1. Assess the quality of fracture reduction and fixation based on the postoperative CT-scans of the ankle, calcaneus, or wrist, determined by a standard scoring protocol

2. Assess the patient relevant outcomes

3. Assess the diagnostic value of an intra-operative 3D-RX-scan in a clinical setting

4. Perform a cost-effectiveness analysis to assess whether the use of 2D-fluoroscopy and the 3D-RX-scan eventually results in cost savings or is compensated for by increased health benefits in comparison with the use of 2D-fluoroscopy only

### Study population

The study population consists of adult patients with a traumatic intra-articular fracture of the wrist, ankle or calcaneus in which operative treatment is indicated.

Inclusion criteria are:

• Adult patients (age > 17 years)

• Distal radius fracture, AO-classification A2-C3, or

• Distal tibial fracture, AO-classification B1-C3, or

• Malleolar fractures, AO-classification A1-C3, or

• Calcaneal fractures, Sanders classificationI-IV

• Fracture surgery (ORIF or CRIF) required (i.e. intra-articular fractures with dislocation).

Only intra-articular fractures will be included in this trial, because the additional value of intra-operative 3D-imaging is to be expected in these types of fractures. The complexity of these fractures warrants more insight in the fracture fragments and fixation material than in extra-articular fractures. It is debatable whether the distal radius fractures AO-classification A2-3 and malleolar fractures C1-3 are true intra-articular fractures. Since we are interested in the congruence of the distal radioulnar joint (DRUJ) and the tibiofibular syndesmosis, these fracture types will be included. Especially in these fracture types, 3D-imaging can give more insight in rotation or subluxation of the ulna or fibula in these articulations.

Exclusion criteria are:

• Pathological fractures, i.e. due to underlying malignant disorder

• Rheumatoid osteoarthritis

• No written informed consent

• Inability to understand trial features due to mental handicap or language problems

• Pregnancy

### Study design

The EF3X-trial is an international randomized multicenter trial, with participation of 4 hospitals (3 university hospitals and 1 regional hospital). Patients eligible for operative treatment of their intra-articular fracture of the wrist, ankle or calcaneus will be randomized after the surgeon is satisfied with the reduction and fixation after 2D-imaging. Patients will be blinded for the availability of the 3D-scan.

When operative treatment is indicated in patients presenting to the Emergency Department (ED) with an intra-articular fracture of the wrist, ankle or calcaneus, they will be counselled and asked for informed consent if the in- and exclusion criteria are met.

**Figure 1:**
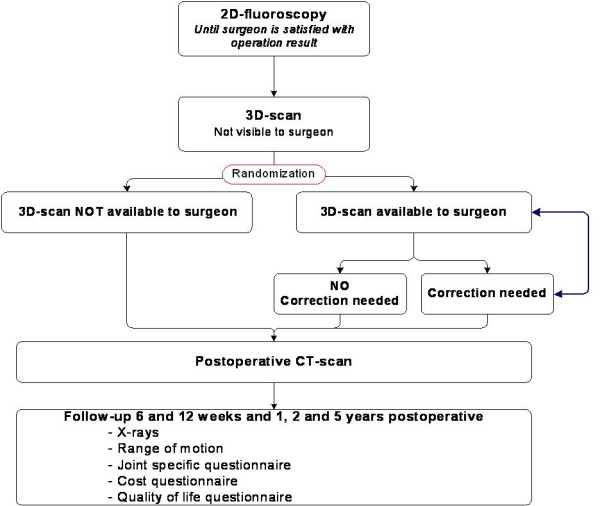
**Flow chart of the EF3X-trial**.

During surgery, initially only 2D-fluoroscopy is used for the intra-operative imaging as part of the usual intra-operative diagnostic procedure (Figure 1). The surgeon will then operate until (s)he is satisfied with fracture reduction and fixation. Then the surgeon will be asked to evaluate the conclusive 2D-images according to the scoring protocol for anatomical reduction and fixation, which is developed in the AMC. After this evaluation a 3D-RX-scan will be performed and randomization will take place.

The randomization will determine whether or not the information of the 3D-scan will be made available to the surgeon. Randomization is performed by an internet randomization module prepared by the AMC Clinical Research Unit. Block randomization is used and randomization will be stratified for the fractured joint (wrist, ankle or calcaneus) and participating centre. Since it is not possible to blind the surgeon, randomization takes place after the surgeon has finished operating based on the information of 2D-fluoroscopy and is ready to close the wound. In this way (s)he cannot anticipate on the likeliness (s)he gets extra information of the 3D-scan. Patients will not be informed about whether or not the 3D-scan was made available to the surgeon.

If the 3D-scan results will not be made available, the surgeon terminates the procedure. If the information of the 3D-scan is available to the surgeon he can act on the findings and, if necessary, surgical corrections can be made. If the surgeon is now satisfied with the operation result conclusive 2D-fluoroscopic images and a conclusive 3D-scan must be performed. The conclusive 3D-scan needs to be evaluated according to the scoring protocol for anatomical reduction. In both randomization groups a CT-scan will be performed postoperatively.

The follow-up visits after hospital discharge will be planned at 6 and 12 weeks and 1, 2 and 5 years postoperatively.

### Primary and secondary endpoints

Our primary outcomes are the quality of fracture reduction and fixation based on the postoperative CT-scans of the wrist, ankle or calcaneus. This will be determined by 3 independent experts. These experts will systematically evaluate the postoperative CT-scans according to a standard scoring protocol. After this evaluation the images will be classified as optimal or suboptimal. A suboptimal reduction and/or fixation will be defined as an indication for a revision following from the systematic evaluation. In addition, this classification will be based on the radiological images alone. Patient related factors, like swelling of the soft tissue will not be taken into account in this evaluation of a (sub)optimal quality of fracture reduction and/or fixation.

A standard scoring protocol for the radiological evaluation will be developed for each joint separately. Although scoring protocols for the wrist, ankle and calcaneus have been described in the literature, most of them lack clinical sensitivity, and are therefore infrequently used [18]. Another reason for not using these scoring protocols is that they merely consist of assessment of distance and angle measurements that have a high interobserver variance and are infrequently used in clinical practice [19-23]. The scoring protocols we developed are based on a Delphi consensus on how to evaluate the different joints. For the wrist and ankle this Delphi consensus was performed in the Netherlands [24]. A Delphi consensus for the calcaneus is currently being performed with international experts.

Second, the patient-relevant outcomes like functional outcome measured by joint specific questionnaires (Patient Rated Wrist Evaluation for the wrist and Foot and Ankle Outcome score and AOFAS for the ankle and calcaneus) will be determined. The patient relevant outcomes one year postoperative will be used as endpoints. Patient relevant outcomes determined at 2 and 5 years postoperatively will be used for a follow up study for the prevalence of posttraumatic osteoarthritis.

Before ending the operation, conclusive 2D-fluoroscopic images and a conclusive 3D-RX-scan are being performed. Together with the postoperative CT-scan (reference test) these radiologic images all represent the final operating result. Therefore the diagnostic value of only the conclusive 2D-fluoroscopic images and 3D-RX-scan will be determined. This will be done for the wrist, ankle and calcaneus separately. Hereby the detection of a suboptimal result, as described above, on the 2D-fluoroscopic images and/or 3D-RX-scan will be compared to the postoperative CT-scans.

A cost-effectiveness analysis will be performed to assess the relative benefit from a societal perspective of the use of the 3D-RX-scan in addition to 2D-fluoroscopy.

Our secondary study outcomes are:

> The number and type of corrections made after 2D-fluoroscopy

> The number and type of corrections made after a 3D-scan

> The number of revision operations within 30 days

> The number and type of complications within 30 days

> The length of the hospital stay expressed in days

> The quality of life measured by the SF-36

### Participating centers

Four centers will enrol patients. Three of these hospitals are Dutch and one University hospital in California is willing to participate. The three Dutch hospitals will consist of 1 regional teaching hospital and two University hospitals. One of the university hospitals, the Academic Medical Center has already started patient recruitment and has included 125 patients in a 15-month period. The other Dutch University hospital, the University Medical Center Utrecht, will start recruiting patients in the summer of 2011 and is also expected to recruit 8-9 patients a month in average. The University hospital in California expects to recruit 50 patients in a one year period. In the regional teaching hospital, the Antonius Hospital in Nieuwegein, approximately 100-150 patients with a wrist, ankle or calcaneus fractures are operated upon yearly. Because this hospital has different locations, for logistic reasons it is not possible to recruit al these patients. It is expected that 75% of these patients will participate in this trial.

### Study outline

#### Recruitment

Patients will be recruited if they are eligible for operative treatment of their intra-articular fracture of the wrist, ankle or calcaneus. This can be at the Emergency Department (ED), the outpatient clinic or the inpatient clinical wards of orthopaedic or trauma surgery. After patients are counselled and informed consent is obtained, they can be included in the study.

#### Intra-operative 3D-scan

For this study the BV Pulsera with 3D-RX (3 Dimensional Rotational X-ray) is used. The BV Pulsera (Philips Healthcare, Best, the Netherlands) consists of a mobile C-arm unit modified to provide a motorized rotational movement and is combined with a Philips 3D-RA workstation. A series of 251 projection images is acquired over a period of 30 seconds during a 200° rotation of the C-arm. The projection images are used to reconstruct a 3D data set. Both volume rendering and slice images are available. The images can be enhanced by colouring the metal present in the joint (Titanview). The radiation exposure of each image in the scanning run is dynamically adjusted to provide the best combination of low dose and optimal image quality. The device is continuously available for the duration of this trial.

#### Radiation dose

Patients with a fractured wrist, ankle, or calcaneus will receive an expected maximum of two 3D-RX-scans, during surgery and two X-rays postoperatively. The maximum equivalent dosage of a 3D-RX-scan of the extremities is 17 μSv. Therefore the additional dosage during the OR of two exams is in the order of 34 μSv. Together with the X-rays performed postoperatively, the radiation dose will approximate 50 μSv. The effective dose of the postoperative CT-exam (120 kV, 150 mAs) will not exceed 0,2 mSv. The total dosage for all radiographic exams performed as part of this trial will therefore be less than 0,25 mSv. A similar effective dose is included in category IIa (0,1-1 mSv) of the ICRP (report ICRP62), which is qualified as a "minor" risk.

### Sample size calculation

Based on the available literature, the frequency of suboptimal fracture reduction is 18-26%. Research in our hospital showed a frequency of 17% (Weide vd, A, Haverlag R, Goslings JC. Inconsistencies in the radiographic analysis of intra-articular fractures. Submitted). We anticipate that a suboptimal fracture reduction and/or fixation will occur in 5% of the patients, when using the 3D-RX-system, as described by Kendoff et al.[25] To be able to detect this difference of 12% using a two-group continuity corrected Chi-square test at an α = 0.05 and a power of 0.80, we will need to include 122 patients per randomization group. To account for an approximately 3% dropout by technical or logistic failures of the 3D-RX-system, a total of 250 patients have to be included for each fracture type.

Because of possibly differential results, patients will be stratified into three groups:

1. Patients with wrist fractures will include distal radius fractures, AO-classification A1-C3

2. Patients with ankle fractures will include distal tibial fractures, AO-classification B1-C3 and malleolar fractures, AO-classification A1-C3

3. Patients with calcaneal fractures will include Sanders classification I-IV

A total of 750 patients will be included in this trial, i.e. 250 for each fracture type.

### Data collection

Pre-operatively baseline data of the patient and fracture type are collected. Intra-operatively the surgeon will be asked to evaluate the operated joint according to the scoring protocol for anatomical reduction and fixation, which is developed in the AMC. If the 3D-scan is available to the surgeon (s)he will evaluate the 3D-scan intra-operatively according to the scoring protocol mentioned above.

During the follow-up visits at 6 and 12 weeks and 1, 2 and 5 years postoperatively the range of motion, functional outcome and strength of the operated joint will be recorded and compared to the contralateral joint. For wrist fractures the 'Patient rated Wrist Evaluation' (PRWE) score will be used, for ankle and calcaneus fractures the 'Foot and Ankle Outcome Score' (FAOS) will be assessed. Quality of life will be determined by the SF-36. These are validated outcome scores. In addition a questionnaire pertaining questions on work-related items and the patients indirect costs of production loss will be assessed.

All intra-operative images (both 2D-fluoroscopy and 3D-scans) and postoperative CT-scans will be evaluated by 3 independent experts in blinded fashion and random order according to a standard scoring protocol. These experts will consist of 2 trauma/orthopedic surgeons and a radiologist. Data collected by the physicians will be entered in a secured Internet module which is specially designed for the EF3X-trial. Patients will be given the choice to receive digital or paper questionnaires. Collection of data and questionnaires will be safeguarded by the trial coordinator.

### Data monitoring

Because of the size of the trial it was considered important to ensure independent review. Therefore a Data Monitoring Committee (DMC) is set up to ensure the safety of the study participants, provide independent review of safety, ensure the integrity of the study conduct and results and review of the formal interim analysis.

### Data analysis

All analyses will be performed in accordance with the intention to treat principle.

The primary outcome, the quality of fracture reduction as well as the quality of fixation will be classified as optimal or suboptimal. This dichotomous outcome, will be described as a percentage in both groups. Differences between study groups will be analysed by means of a Chi-square test. A p-value < 0.05 will be taken as the threshold for statistical significance.

The scores of the functional outcomes determined by joint specific questionnaires at 1 year postoperative will be expressed as means and standard deviations (SD) in case of a normal distribution. Non-normally distributed outcomes will be expressed as medians and interquartile ranges. Normality of continuous data will be tested with the Shapiro-Wilk and Kolmogorov-Smirnov test and by inspecting the frequency distributions (histograms). The homogeneity of variances will be tested using the Levene's test. The functional outcomes will be assessed using the Student's T-test (parametric data) or the Mann-Whitney *U*-test (non-parametric data). Differences will be considered statistically significant when P-values are < 0.05.

The diagnostic accuracy of a suboptimal quality of fracture reduction and/or fixation will be determined for reduction and fixation for the wrist, ankle and calcaneus separately. Sensitivity, specificity, positive and negative predictive values for both 2D-fluoroscopy and the 3D-RX-scan will be calculated with the corresponding 95% confidence intervals for the classification of an optimal or suboptimal result as described above. This will be determined for both 2D-fluoroscopy and the 3D-RX-scan with the postoperative CT-scan as reference standard.

The number of patients in which corrections were performed intra-operatively after 2D-fluoroscopy or the 3D-RX-scan will be described as percentages. Differences between study groups will be analysed by means of a Chi-square test. The same analysis will be done for the number of patients with a revision operation or complication within 30 days. The length of hospital stay will be expressed as medians and interquartile ranges, and differences between groups will be analysed using a Mann-Whitney U test. The quality of life measured by the SF-36 will be expressed as means and standard deviations (SD) after testing for normal distribution and compared between groups using an unpaired Student t-test. For all secondary parameters a p-value < 0.05 will be taken as the threshold for statistical significance.

### Economic evaluation

The economic evaluation of intra-operative use of 3D-RX against the use of 2D-fluoroscopy as its best alternative will be performed from a societal perspective as both, a cost-effective and a cost-utility analysis. As the costs of a 3D-RX-system are 1,5 times the costs of a standard C-arm and there is the risk of over diagnosing, an economic evaluation is warranted. This cost-effectiveness analysis is chosen to comply with the clinical endpoint and enables assessment of diagnostic strategies and therapeutic interventions within the field of joint trauma care. The cost-utility analysis is chosen to enable comparisons between the currently proposed optimization of health care (3D versus 2D imaging) on the one hand and new developments and technologies for other diseases and in other areas of medicine on the other hand. The primary outcomes will be the costs per patient with optimal fracture reduction (at 12 weeks post index operation, thus including assessments of re-interventions) and the costs per QALY respectively. The time horizon will be 12 weeks following the index operation. With this length of the follow-up period no discounting of costs will take place.

Incremental cost-effectiveness and cost-utility ratios will be calculated for the extra costs per extra patient with optimal fracture reduction and the extra costs per QALY gained. Univariable and multivariable sensitivity analyses will be applied for unit costs of 3D-scan and country-specific health utility value sets (see below). Bias corrected and accelerated bootstrapping will be done to account for sampling variability. All analyses will be done for each subgroup of patients (with wrist, ankle or calcaneus fracture respectively).

The costs will include the direct medical and non-medical as well as the indirect non-medical costs of care. The direct medical costs include the costs of diagnostics, surgery, in-patient stay, and out-of-hospital care postdischarge (e.g. family practitioner, physiotherapist, rehabilitation care). Also, out-of-pocket expenses of patients will be quantified (over-the-counter medication, private help at home, etc.). The indirect non-medical costs of production loss due to work absenteism will also be calculated. Principally, the friction cost method will be applied to quantify these production losses (in practice though, the length of the friction period at the time of analysis presumably will be longer than the planned follow-up period of patients). Volume data on health care resource use, out-of-pocket expenses and work absenteism will be gathered with case report forms, hospital information systems, and a patient questionnaire, partially based on the Health and Labour questionnaire [26]. Unit costs will derive from the national guideline on costing in health care research [27]. Unit costs will be price-indexed to derive cost estimates for the base year 2011.

Fractures can be quite disabling in daily life. In addition to the already mentioned general (SF-36) and domain-specific (FAOS, PRWE) quality of life questionnaires, the EQ-5D will be applied as a health utility instrument for use in the cost-utility analysis. The health status scoring profiles gathered with the EQ-5D will be transformed into health utilities using available time trade-off based valuation algorithms from the literature. Initially, the Dutch valuations will be used [28]. In a sensitivity analysis the internationally more frequently applied algorithm from the UK will be applied [29].

### Early experience from the ongoing trial

During a period of 15 months 125 patients have been included in one hospital alone. Next to recruitment of patients in the Academic Medical Center (AMC), recruitment will also start in the St. Antonius hospital in Nieuwegein and the University Medical Center Utrecht in the Netherlands. Internationally the University of California San Francisco Medical Center will participate. Patient recruitment in the AMC has gone according to expectations and there are few patients not willing to participate. Due the acute nature of fracture surgery some patients are not able to be counselled and therefore excluded from participation.

For the clinical follow up it appears that 6 weeks postoperative is too early to fill in the selfreported joint-specific questionnaires. Most patients have had 6 weeks of cast immobilisation and have not performed the activities questioned or were advised not to perform some of the activities. For this reason most patients leave some answers blank. Most of the questionnaires filled in after 12 weeks and 1 year postoperatively are filled in correctly and will be used as an endpoint for the clinical outcome.

## Discussion

Intra-operative 3D-imaging in fractures of the extremities has been introduced a decade ago. Several cadaver studies have been performed to determine the diagnostic value of 3D-imaging, showing that it is comparable to CT-scanning. Clinical studies have shown that extra corrections in 11-30% are performed when using additional 3D-imaging during fracture surgery. Still the effectiveness of the corrections mentioned before on the quality of fracture reduction and fixation and patient relevant outcomes has not yet been determined. The EF3X-trial aims to provide evidence based answers on the effectiveness of the intra-operative use of 3D-imaging intra-articular fractures of the extremities.

This trial compares the use of additional 3D-imaging in surgical treatment of intra-articular fractures of the extremities. Although it is widely accepted to strive for anatomical fracture reduction and fixation, there's still little evidence to support this. Therefore, in addition to the short-term radiological endpoint, also the long-term patient relevant outcomes will be determined in this trial.

## Competing interests

M.S.H. Beerekamp is supported by an unrestricted grant from Philips Healthcare, Best, the Netherlands

## Authors' contributions

MSB drafted the manuscript. DTU, MD, NWS and JCG co-authored the writing of the manuscript. JCG is the principal investigator. All other authors participated in the design of the study during several meetings and are local investigators at the participating centers. All authors edited the manuscript and read and approved the final manuscript.

## Pre-publication history

The pre-publication history for this paper can be accessed here:

http://www.biomedcentral.com/1471-2474/12/151/prepub
